# Fatigue is Highly Prevalent in Patients with Asthma and Contributes to the Burden of Disease

**DOI:** 10.3390/jcm7120471

**Published:** 2018-11-23

**Authors:** Maarten Van Herck, Martijn A. Spruit, Chris Burtin, Remco Djamin, Jeanine Antons, Yvonne M. J. Goërtz, Zjala Ebadi, Daisy J. A. Janssen, Jan H. Vercoulen, Jeannette B. Peters, Melissa S. Y. Thong, Jacqueline Otker, Arnold Coors, Mirjam A. G. Sprangers, Jean W. M. Muris, Emiel F. M. Wouters, Alex J. van ’t Hul

**Affiliations:** 1 REVAL Rehabilitation Research Center, BIOMED Biomedical Research Institute, Faculty of Rehabilitation Sciences, Hasselt University, 3590 Diepenbeek, Belgium; martijnspruit@ciro-horn.nl (M.A.S.); chris.burtin@uhasselt.be (C.B.); 2 Department of Research and Education, CIRO, Centre of Expertise for Chronic Organ Failure, 6085 NM Horn, The Netherlands; yvonnegoertz@ciro-horn.nl (Y.M.J.G.); daisyjanssen@ciro-horn.nl (D.J.A.J.); ewouters@ciro-horn.nl (E.F.M.W.); 3 Department of Respiratory Medicine, Maastricht University Medical Center (MUMC+), 6229 HX Maastricht, The Netherlands; 4 NUTRIM School of Nutrition and Translational Research in Metabolism, 6229 ER Maastricht, The Netherlands; 5 Department of Respiratory Medicine, Amphia Hospital, 4818 CK Breda, The Netherlands; rdjamin@amphia.nl; 6 Department of Pulmonary Medicine, Radboud University Medical Center (Radboudumc), 6525 GA Nijmegen, The Netherlands; jeanine.antons@radboudumc.nl (J.A.); alex.vanthul@radboudumc.nl (A.J.v.H.); 7 Department of Medical Psychology, Radboudumc, 6525 GA Nijmegen, The Netherlands; zjala.ebadi@radboudumc.nl (Z.E.); jan.vercoulen@radboudumc.nl (J.H.V.); jeannette.jacobs-peters@radboudumc.nl (J.B.P.); 8 Centre of expertise for palliative care, MUMC+, 6229 HX Maastricht, The Netherlands; 9 Department of Medical Psychology, Amsterdam University Medical Centers, location AMC, 1105 AZ Amsterdam, The Netherlands; s.y.thong@amc.uva.nl (M.S.Y.T.); m.a.sprangers@amc.uva.nl (M.A.G.S.); 10 Member of Lung Foundation Netherlands, 3818 LE Amersfoort, The Netherlands; jmotker@telfort.nl; 11 Member of Patient Advisory Board, Radboudumc, 6525 GA Nijmegen, The Netherlands; arnoldcoors@mac.com; 12 Department of General Practice, MUMC+, 6229 HX Maastricht, The Netherlands; jean.muris@maastrichtuniversity.nl

**Keywords:** asthma, fatigue, quality of life

## Abstract

The 2018 update of the Global Strategy for Asthma Management and Prevention does not mention fatigue-related symptoms. Nevertheless, patients with asthma frequently report tiredness, lack of energy, and daytime sleepiness. Quantitative research regarding the prevalence of fatigue in asthmatic patients is lacking. This retrospective cross-sectional study of outpatients with asthma upon referral to a chest physician assessed fatigue (Checklist Individual Strength-Fatigue (CIS-Fatigue)), lung function (spirometry), asthma control (Asthma Control Questionnaire (ACQ)), dyspnea (Medical Research Council (MRC) scale), exercise capacity (six-minute walk test (6MWT)), and asthma-related Quality-of-Life (QoL), Asthma Quality of Life Questionnaire (AQLQ) during a comprehensive health-status assessment. In total, 733 asthmatic patients were eligible and analyzed (47.4 ± 16.3 years, 41.1% male). Severe fatigue (CIS-Fatigue ≥ 36 points) was detected in 62.6% of patients. Fatigue was not related to airflow limitation (FEV1, *ρ* = −0.083); was related moderately to ACQ (*ρ* = 0.455), AQLQ (*ρ* = −0.554), and MRC (*ρ* = 0.435; all *p*-values < 0.001); and was related weakly to 6MWT (*ρ* = −0.243, *p* < 0.001). In stepwise multiple regression analysis, 28.9% of variance in fatigue was explained by ACQ (21.0%), MRC (6.5%), and age (1.4%). As for AQLQ, 42.2% of variance was explained by fatigue (29.8%), MRC (8.6%), exacerbation rate (2.6%), and age (1.2%). Severe fatigue is highly prevalent in asthmatic patients; it is an important determinant of disease-specific QoL and a crucial yet ignored patient-related outcome in patients with asthma.

## 1. Introduction

Wheezing, breathlessness, chest tightness, and cough are cardinal symptoms in patients with asthma. These symptoms vary over time and are often triggered by exercise, emotions, dust, and/or exposure to allergens [1]. In addition to these well-known respiratory symptoms, asthmatic patients also report that they experience tiredness [2,3], lack of energy [3], and daytime sleepiness [4]. Thus, fatigue, defined as the subjective feeling of tiredness or exhaustion [5], may be a common and clinically relevant symptom in asthmatic patients. Indeed, severe fatigue was present in 90% of highly-selected patients with severe and uncontrolled asthma referred to a tertiary-care, high-altitude pulmonary rehabilitation program [6]. Besides that, fatigue was significantly correlated with disease-specific quality of life (QoL) [6]. To date, the prevalence of severe fatigue and its association with clinically relevant features in a broader, more general sample of patients with asthma remains unknown.

Therefore, the aim of the present study was (1) to assess the prevalence of severe fatigue in a general sample of patients with asthma upon referral to a chest physician for an outpatient consultation, (2) to assess the independent determinants of fatigue, and (3) to assess whether and to what extent fatigue is an independent determinant of disease-specific QoL.

## 2. Methods

### 2.1. Study Design and Participants

In this retrospective, observational, cross-sectional study, all participants underwent a comprehensive health status assessment between April 2014 and April 2018. The assessment was part of usual outpatient care in the Amphia Hospital in Breda and the Radboud University Medical Centre (Radboudumc) in Nijmegen (both in The Netherlands). General practitioners (GP) referred these patients for the first time to the outpatient consultation of a chest physician in a secondary care setting because of persistent complaints with an unsatisfactory response to the treatment offered in primary care. Eligibility criteria were diagnosis of asthma (Global Initiative for Asthma, GINA) [1] and data available regarding gender, age, fatigue, and lung function. Patients with an asthma exacerbation in the previous three months (clinical unstable patients) and/or aged <18 years were excluded. The Medical Ethical Committee of the Radboudumc approved this retrospective study of data collected during usual care (MEC-number: 2018-4357).

### 2.2. Health Status and Disease-Specific Characteristics

The following data were systematically registered: age, gender, weight, height, waist circumference, current smoking status, employment status, and education level. Body Mass Index (BMI; kg/m^2^) was calculated and classified according to WHO guidelines [7].

Fatigue severity was measured by the subjective fatigue subscale of the Checklist Individual Strength-Fatigue (CIS-Fatigue) [8]. The CIS-Fatigue is a standardized and validated questionnaire that has been used in healthy subjects [9,10,11] and among various patient populations [11]. The subscale regarding subjective fatigue consists of eight items scored on a seven-point Likert scale. The scores range from 8 to 56 points. A score of ≤26 points indicates normal fatigue, scores between 27 and 35 indicate mild fatigue, and a score of ≥36 indicates severe fatigue [8].

Disease-specific QoL was scored using the Asthma Quality of Life Questionnaire (AQLQ) [12], with scores ranging from 1 (‘severely impaired’) to 7 points (‘not impaired’). Asthma control was scored using the Asthma Control Questionnaire (ACQ). Scores range between 0 and 6 points, and can be classified into three categories: controlled (≤0.75), partially controlled (0.76–1.49), and uncontrolled asthma (≥1.5) [13]. Pre- and post-bronchodilator lung function measurements were completed and are expressed as a percentage of the Global Lung Function Initiative reference values [14]. The presence of comorbidities was scored using the Charlson Comorbidity Index (CCI) [15], whereas exercise capacity was measured using the six-minute walking test (6MWT) according to European Respiratory Society/American Thoracic Society Technical Standard [16,17]. The degree of dyspnea was scored using the Medical Research Council (MRC) scale, and the number of asthma exacerbations and hospitalizations during the past 12 months were recorded.

### 2.3. Statistical Analysis

All statistical analyses were conducted using SPSS v.25.0 (International Business Machines Corp., Armonk, NY, USA). Data were presented as mean ± standard deviation (SD), median and interquartile range, or frequencies and proportions, as appropriate. Differences between groups for continuous data were analyzed by one-way analysis of variance or the non-parametric pendant (Kruskall-Wallis test); if significant, a pairwise post-hoc test (unpaired t-test or Mann-Whitney U test, respectively) was performed and significant values were adjusted by Bonferonni correction for multiple comparison. Categorical data were analyzed with the Chi-square test or Fisher Exact test. Correlations were assessed by Pearson or Spearman’s rank correlation where appropriate. Stepwise multiple linear regression models were developed to explain the variance in fatigue and disease-specific QoL. Based upon results of bivariate regression analysis between independent variables and fatigue or AQLQ, respectively, significant correlates were selected. If multicollinearity was present in the model (Variance Inflation Factor, VIF > 5), variables with VIF > 5 were identified [18] and removed from the model. The level of significance was set at 0.01 for all statistical tests (two-tailed). 

## 3. Results

### 3.1. Patient Characteristics

**Figure 1:**
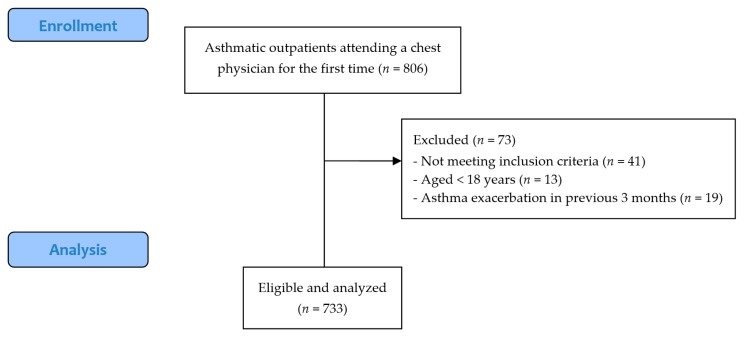
Flowchart of participants’ inclusion. *n*: number of subjects.

Eight hundred and six outpatients with asthma attended a chest physician for the first time and underwent a comprehensive health status assessment. In total, 733 asthmatic patients from the Amphia hospital and Radboudumc (respectively, *n* = 560 and *n* = 173) were eligible and analyzed. Reasons for exclusion were not meeting inclusion criteria (*n* = 41 in total) due to the absence of data regarding fatigue and lung function (respectively, *n* = 12 and *n* = 29), being aged <18 years (*n* = 13), and having had an asthma exacerbation in the previous three months (*n* = 19). The flowchart of participants’ inclusion is illustrated in Figure 1.

Eighteen percent of the patients had a CCI total score of ≥1 point, indicating the presence of ≥1 comorbidity. Twenty-six percent had partially controlled asthma, while 60% had uncontrolled asthma. Forty-three percent of the patients had an MRC dyspnea grade ≥3, indicating functional impairment during minimal effort or activities of daily living due to dyspnea. All details can be found in Table 1.

### 3.2. Prevalence of Fatigue

Patients had a mean fatigue score of 38.4 ± 12.4 points. 18.8% of the patients reported normal fatigue, 18.6% mild fatigue and 62.6% severe fatigue.

### 3.3. Differences between Asthmatic Patients with Normal, Mild, and Severe Fatigue

**Figure 2:**
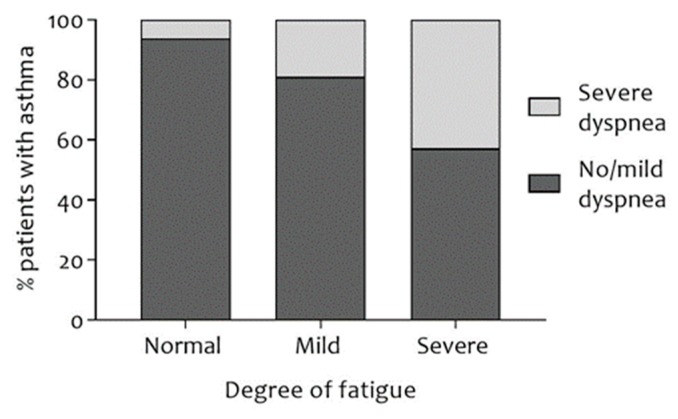
Proportion of asthmatic patients with normal/mild dyspnea (MRC < 3) or severe dyspnea (MRC ≥ 3) after stratification for the degree of fatigue. A statistically significant association between degree of fatigue and degree of dyspnea was observed, *χ*^2^ (2, *n* = 532) = 56.229, *V* = 0.325, and *p* < 0.001. MRC: Medical Research Council; *n*: number of subjects; *V*: Cramer’s V.

**Figure 3:**
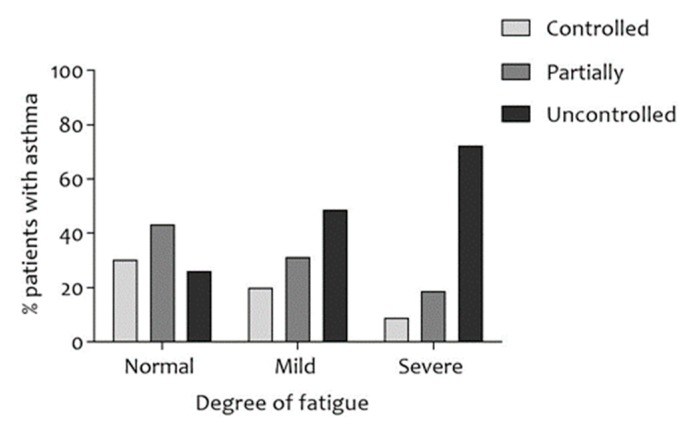
Proportion of asthmatic patients with controlled (ACQ ≤ 0.75), partially controlled (ACQ 0.76–1.49), and uncontrolled asthma (ACQ ≥ 1.5) after stratification for the degree of fatigue. A statistically significant association between degree of fatigue and degree of asthma control was observed; *χ*^2^ (4, *n* = 664) = 93.073, *V* = 0.265, and *p* < 0.001. ACQ: Asthma Control Questionnaire; *n*: number of subjects; *V*: Cramer’s V.

Patients with severe fatigue were more likely to be female and younger and were more likely to be obese, more likely to have had ≥1 exacerbations in the last 12 months, had a lower educational level, a worse exercise capacity, lower forced vital capacity (FVC), more dyspnea (Figure 2), a worse disease-specific QoL, and worse asthma control (Figure 3; all *p* < 0.01). Details can be found in Table 1.

### 3.4. Correlations and Determinants of Fatigue

**Figure 4:**
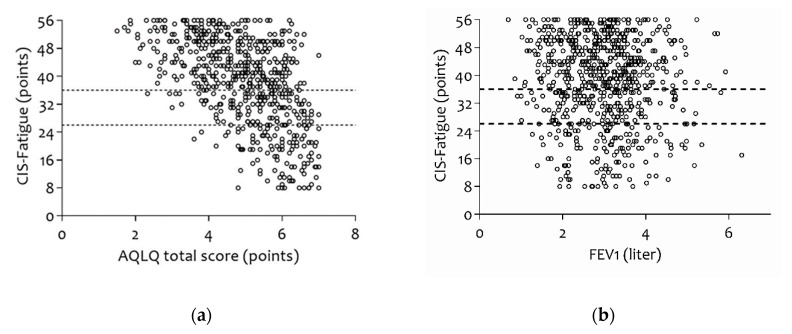
(**a**) Correlation between fatigue and asthma-related quality of life (*ρ* = −0.554, *p* < 0.001) and (**b**) absence of correlation between fatigue and FEV1 (*ρ* = −0.083, *p* = 0.025). AQLQ: Asthma Quality of Life Questionnaire; CIS-Fatigue: Checklist Individual Strength-Fatigue; and FEV1: Forced Expiratory Volume in the first second.

Table S1 in the online data supplement gives an overview of all correlations between the CIS-Fatigue score and clinical traits. Significant correlations were found for gender (male, *ρ* = 0.139), age (*ρ* = −0.115), BMI (*ρ* = 0.132), asthma exacerbations in the last 12 months (*ρ* = 0.200), level of education (secondary general education or higher, *ρ* = 0.145), 6MWT (in m, *ρ* = −0.243), FVC (in liter, *ρ* = −0.158), Tiffeneau-index (*ρ* = 0.133), MRC-Dyspnea (*ρ* = 0.435), AQLQ (total score: *ρ* = −0.554, Figure 4a; and all subdomains, between *ρ* = −0.345 and *ρ* = −0.591), and ACQ (*ρ* = 0.455). No significant relationship between fatigue and Forced Expiratory Volume in one second (FEV1) was found (*ρ* = −0.083, *p* = 0.025, Figure 4b).

Stepwise multiple regression model explained 28.9% of variance in CIS-Fatigue, of which ACQ (21.0%), MRC dyspnea grade (6.5%), and age (1.4%) were significant predictors, adjusted *R*^2^ = 0.289, F (3, 378) = 52.512, and *p* < 0.001. Details can be found in Table S2 (online data supplement).

### 3.5. Correlations and Determinants of Disease-Specific QoL

Table S3 in the online data supplement gives an overview of all correlations between the total AQLQ score and clinical traits. Significant correlations were found for age (*ρ* = 0.115), BMI (*ρ* = −0.194), asthma exacerbations in the last 12 months (*ρ* = −0.279), level of education (secondary general education or higher, *ρ* = −0.156), CIS-Fatigue (*ρ* = −0.554), 6MWT (in m, *ρ* = 0.287), FVC (in liter, *ρ* = 0.161), Tiffeneau-index (*ρ* = −0.162), MRC-Dyspnea (*ρ* = −0.488), and ACQ (*ρ* = −0.777).

As AQLQ and ACQ measure a similar concept, a strong correlation between ACQ and AQLQ total score was found (*ρ* = −0.777) [19]. ACQ was left out of the multiple regression analysis of AQLQ. The stepwise multiple regression model explained 42.2% of variance in AQLQ total score, adjusted *R*^2^ = 0.422, F (4, 377) = 70.585, and *p* < 0.001. Significant predictors of asthma-related QoL were CIS-Fatigue (29.8%), MRC dyspnea grade (8.6%), asthma exacerbations in the last 12 months (2.6%), and age (1.2%). Details can be found in Table S4 (online data supplement).

## 4. Discussion

The present study is the first large-scale, multi-center, cross-sectional study of fatigue in outpatients with asthma. Severe fatigue is present in about two-thirds of the patients, and fatigue independently determines disease-specific QoL.

Qualitative research suggested that patients with asthma suffer from fatigue [2,3], which was quantified by Peters and colleagues who identified severe fatigue in 90% of their sample of patients referred to tertiary-care using the CIS-Fatigue [6]. In the current study, the proportion of patients with severe fatigue (63%) is lower but clearly higher than the 10% prevalence as observed in healthy elderly [20,21], and in accordance with other chronic non-communicable diseases, such as chronic obstructive pulmonary disease (COPD) (49%), chronic renal failure (42–89%) [22], and chronic heart failure (39%) [23]. The discordance with Peters and colleagues can most probably be explained by the fact they studied patients with severe and uncontrolled asthma referred to a tertiary-care, high-altitude pulmonary rehabilitation program [6], while in the current study a broad sample of asthmatic patients was studied.

ACQ and MRC dyspnea grade were found to be significantly different among levels of fatigue. Indeed, the higher the level of fatigue, the higher the proportion of patients with severe dyspnea and uncontrolled asthma (respectively, Figure 2 and Figure 3). A possible explanation for this finding may be a more sedentary behavior due to fatigue (or vice versa) [20,24,25,26], whereas physical inactivity is associated with poor asthma control and high MRC dyspnea grade [27]. Unfortunately, this could not be confirmed, since data regarding steps per day, sedentary time, and physical activity was unavailable. Asthmatic patients with severe fatigue were more likely to be female, younger, less educated, and to have a lower exercise capacity. These finding are in accordance with recent studies in the general population [20] and patients with COPD [21].

Only one-third (28.9%) of the variance in CIS-Fatigue scores can be explained by ACQ scores, MRC dyspnea grade, and age. Similar results were found in COPD [21]. This suggests that the underlying causes of fatigue are multifactorial [5], and other physical, psychological, behavioral and/or systemic factors, which may play a role in precipitating and/or perpetuating fatigue in patients with asthma, should be considered in future studies as well as in clinical care, and/or systemic factors should be considered in future studies as well as in clinical care [28]. Interestingly, spirometry-derived attributes were not maintained in the multiple regression model. Therefore, it seems reasonable to conclude that the degree of lung function impairment is not useful to estimate the degree of fatigue in asthmatic patients. Moreover, these results may also suggest that conventional respiratory drug therapy may have limited effects on reducing fatigue in asthmatic patients. Indeed, ‘tiredness’ is the most common patient-reported adverse event following asthma treatment [29]. Future research needs to record pharmacological therapy and disease duration to assess the impact of both variables on symptom burden, and fatigue in particular.

Fatigue is the most important predictor of AQLQ. Indeed, fatigue alone explains 29.8% of the variance, while MRC dyspnea grade explains only an additional 8.6%. This indicates that fatigue has a significant influence on disease-specific QoL in asthmatic patients and that disease-specific QoL most probably increases by tackling fatigue. In patients with COPD, pulmonary rehabilitation programs show an additional effect above conventional (pharmacological) therapy on dyspnea and fatigue [30]. Exercise therapy for asthmatic patients is safe (under certain conditions) [1,31] and beneficial, reducing asthma-symptoms and exacerbation rate [32]. There is no evidence regarding exercise therapy affecting subjective fatigue in patients with asthma. A recent study demonstrated significant improvements in persons with prolonged fatigue who reported fatigue to be one of their major health complaints after completion of a six week-long structured exercise program [33].

### 4.1. Methodological Considerations

Some methodological considerations warrant attention. The large sample consisted of patients with asthma referred by the GP to the chest physician for an outpatient consultation. This resulted in a broad and heterogeneous sample of asthmatic patients with controlled, partially controlled, or uncontrolled asthma. This extends the generalizability of the current results. However, all patients with an exacerbation/hospitalization within three months of the study were ineligible to participate, as fatigue may worsen during an exacerbation [1,34]. Literature suggests that a recent exacerbation has an important adverse impact on physical activity, QoL [35], and fatigue [28,34,36]. Therefore, it is possible that the current findings concerning the prevalence of severe fatigue (in asthmatic patients referred to secondary care) are an underestimation of the actual prevalence of fatigue in a population with both clinically stable and unstable asthma. Fatigue was measured by CIS-Fatigue, which assesses overall fatigue during the past two weeks, based on patients’ recall, which may be subject to bias. Additionally, this approach does not allow one to assess possible day-to-day variability in symptoms, whereas fatigue is expected to fluctuate over time. More detailed measurement of fatigue requires repeated measurements during daytime and over a more extended period of time. Future studies may want to consider using ecological momentary assessment to better understand these patterns, get more detailed information, and overcome recall [37].

Multiple physical, psychological, behavioral, and/or systemic factors, including symptoms of anxiety and depression, were not available in the current study. However, these factors may explain, at least in part, the variance in fatigue as observed in patients with asthma.

### 4.2. Clinical Implications

Fatigue as a debilitating symptom of patients with asthma is not considered in the 2018 update of *GINA* [1]. The 2018 update of *GINA* only mentions fatigue in the context of hypercapnia and controlled oxygen therapy during the management of an asthma exacerbation [1]. The current findings suggest that fatigue is an important patient-reported outcome in patients with asthma. Indeed, it is highly-prevalent and a strong determinant of disease-specific QoL, which makes it an important target for treatment. Our study also emphasizes the importance of the use of short, reliable fatigue questionnaires such as CIS-Fatigue, as part of the general health status assessment.

The current findings suggest that fatigue must be assessed and handled in primary and secondary asthma care, whereas fatigue is an important determinant of patient-reported, disease-specific QoL. Besides that, asthma care beyond the GP should be considered much earlier in patient’s disease trajectory. Indeed, despite practice-based GP-guided pharmacological therapy (and education), these patients still suffer from severe dyspnea (43%), severe fatigue (63%), being overweight or obese (40% or 27%, respectively), reduced exercise capacity (74%), and/or uncontrolled asthma (60%). Therefore, asthma care must not only focus on respiratory symptoms in the future but also non-respiratory symptoms such as fatigue, exercise intolerance, and asthma control.

## 5. Conclusions

To conclude, fatigue is highly prevalent in patients with asthma and moderately associated with dyspnea, asthma control, and asthma-related QoL. Besides, fatigue is poorly and non-significant associated with the degree of lung function impairment. Moreover, disease-specific QoL is partly determined by fatigue, and this in turn makes it an important patient-related outcome in patients with asthma.

## Figures and Tables

**Table 1:** General characteristics of patients with asthma (*n* = 733) stratified for fatigue severity in normal, mild, or severe fatigue based on the Checklist Individual Strength-Fatigue (CIS-Fatigue) questionnaire.

Variables	Patients with Asthma (*n* = 733)	Fatigue Severity in Asthmatic Patients			*p*-Value
		Normal Fatigue (*n* = 138)	Mild Fatigue (*n* = 136)	Severe Fatigue (*n* = 459)	
CIS-Fatigue	38.4 ± 12.4	18.5 ± 5.6	31.6 ± 2.6 *	46.4 ± 6.1 *^†^	<0.001
General Characteristics					
Gender (male, %)	302 (41.2)	78 (56.5)	55 (40.4)	169 (36.8)	<0.001
Age (years)	47.4 ± 16.3	50.4 ± 16.4	48.5 ± 17.0	46.1 ± 15.9	0.009
Weight (kg)	80.7 ± 17.7	81.6 ± 20.1	78.7 ± 16.8	81.1 ± 17.2	0.418
BMI (kg/m^2^)	27.5 ± 5.5	26.5 ± 4.9	26.8 ± 4.9	28.0 ± 5.8	0.019
Underweight (n, %)	11 (1.5)	1 (0.7)	4 (2.9)	6 (1.3)	0.005
Normal (n, %)	242 (33.0)	55 (39.9)	42 (30.9)	145 (31.6)	
Overweight (n, %)	286 (39.8)	57 (41.3)	64 (47.1)	165 (35.9)	
Obese (n, %)	194 (26.5)	25 (18.1)	26 (19.1)	143 (31.2)	
Waist circumference ^a^ (cm)	97.5 ± 14.9	96.3 ± 13.7	96.7 ± 14.4	98.2 ± 15.4	0.474
Exacerbations last 12 months ^b^ (n)	0 (0–2)	0 (0–1)	0 (0–1)	0 (0–2) *	<0.001
Patients with ≥1 exacerbations last 12 months (n, %)	281 (44.5)	33 (28.0)	50 (45.5)	198 (49.1)	<0.001
Hospitalization last 12 months ^c^ (n)	0 (0–0)	0 (0–0)	0 (0–0)	0 (0–0)	0.344
Patients with ≥1 hospitalization last 12 months (n, %)	18 (3.0)	1 (0.9)	4 (3.8)	13 (3.4)	0.326
Smoking status ^d^ Current smoker (n,%)	164 (24.0)	24 (19.5)	25 (19.7)	115 (26.5)	0.126
Level of education ^e^ Secondary general education or higher (n,%)	295 (43.0)	68 (51.5)	62 (51.2)	165 (38.1)	0.003
Employment status ^f^ Paid work (n, %)	413 (61.1)	76 (61.3)	70 (56.9)	267 (62.2)	0.566
Exercise Capacity					
6MWT ^g^ (m)	519.1 ± 109.2	565.2 ± 93.8	525.8 ± 89.0	503.5 ± 114.6 *	<0.001
6MWT ^g^ (% predicted)	71.9 ± 13.8	79.4 ± 12.4	74.0 ± 11.1 *	69.1 ± 14.1 *^†^	<0.001
Patients with <80% predicted ^g^ (n, %)	481 (73.8)	62 (50.4)	82 (70.7)	337 (81.6)	<0.001
Spirometry					
FEV1 (% predicted)	87.7 ± 17.4	88.3 ± 15.7	88.1 ± 19.3	87.3 ± 17.4	0.867
FEV1 (liter)	3.0 ± 0.9	3.1 ± 1.0	2.9 ± 0.9	2.9 ± 0.9	0.203
FVC (% predicted)	98.7 ± 14.2	102.2 ± 14.2	100.4 ± 14.2	97.1 ± 14.0 *	0.001
FVC (liter)	4.2 ± 1.2	4.5 ± 1.3	4.2 ± 1.1	4.0 ± 1.1 *	<0.001
FEV1/VC * 100 (%)	71.6 ± 11.9	69.1 ± 11.0	70.3 ± 11.9	72.8 ± 12.0 *	<0.001
Comorbidities					
CCI ^h^ (p)	0 (0–0)	0 (0–0)	0 (0–0)	0 (0–0)	0.200
Persons with ≥1 comorbidities ^h^ (n, %)	70 (17.6)	8 (11.0)	14 (18.4)	48 (19.4)	0.249
Dyspnea					
MRC-Dyspnea ^i^ (p)	2 (1–3)	1 (1–2)	2 (1–2) *	2 (2–3) *^†^	<0.001
MRC grade ≥ 3 (severe dyspnea) ^i^ (n, %)	168 (31.6)	6 (6.1)	19 (19.0)	143 (42.8)	<0.001
Disease-Specific QoL					
AQLQ ^j^ (p)	4.9 ± 1.3	5.9 ± 0.7	5.3 ± 1.0 *	4.5 ± 1.1 *^†^	<0.001
Symptoms ^j^ (p)	4.7 ± 1.3	5.7 ± 0.9	5.1 ± 1.2 *	4.4 ± 1.2 *^†^	<0.001
Activity limitation ^j^ (p)	4.7 ± 1.5	6.1 ± 1.0	5.2 ± 1.2 *	4.1 ± 1.4 *^†^	<0.001
Emotional function ^j^ (p)	5.3 ± 1.4	6.2 ± 0.8	5.6 ± 1.2	5.0 ± 1.5 *^†^	<0.001
Environmental exposure ^j^ (p)	5.2 ± 1.5	5.9 ± 1.1	5.6 ± 1.3	4.9 ± 1.5 *^†^	<0.001
Asthma Control					
ACQ ^k^ (p)	1.8 ± 1.0	1.2 ± 0.7	1.6 ± 0.8 *	2.1 ± 1.0 *^†^	<0.001
Partially controlled asthmatic patients (n, %)	170 (25.6)	53 (43.4)	39 (31.2)	78 (18.7)	<0.001
Uncontrolled asthmatic patients (n, %)	395 (59.5)	32 (26.2)	61 (48.8)	302 (72.4)	

Data is presented as mean ± SD, median (IQR), or number (%). *p*-value in bold indicates a significant difference. * indicates significantly different from normal fatigue. ^†^ indicates significant difference between mild and severe fatigue. Alphabetic characters in superscript indicates a sample size deviant from *n* = 733 with the following: a. *n* = 696 (normal, mild, and severe resp. 131, 132, and 433), b. *n* = 631 (normal, mild, and severe resp. 118, 110, and 403), c. *n* = 601 (normal, mild, and severe resp. 112, 106, and 383), d. *n* = 684 (normal, mild, and severe resp. 123, 127, and 434), e. *n* = 686 (normal, mild, and severe resp. 132, 121, and 433), f. *n* = 676 (normal, mild, and severe resp. 124, 123, and 429), g. *n* = 652 (normal, mild, and severe resp. 123, 116, and 413), h. *n* = 397 (normal, mild, and severe resp. 73, 76, and 248), i. *n* = 532 (normal, mild, and severe resp. 98, 100, and 334), j. *n* = 653 (normal, mild, and severe resp. 123, 122, and 408), and k. *n* = 664 (normal, mild, and severe resp. 122, 125, and 417). Abbreviations: AQLQ: Asthma Quality of Life Questionnaire; ACQ: Asthma Control Questionnaire; BMI: Body Mass Index; CIS-Fatigue: Checklist Individual Strength-Fatigue; CCI: Charlson Comorbidity Index; FEV1: Forced Expiratory Volume in one second; FEV1/VC * 100: Tiffeneau index; FVC: Forced Vital Capacity; *n*: number of subjects; p: points; MRC-Dyspnea: Medical Research Council-Dyspnea; QoL: Quality of life; 6MWT: Six-Minute Walk Test.

## Supplementary Materials

The following are available online at http://www.mdpi.com/2077-0383/7/12/471/s1, Table S1: Spearman rank correlation coefficients of demographical, health status and disease-specific characteristics with fatigue in asthmatic patients (*n* = 733), Table S2: Multiple stepwise regression analysis of the significant variables associated with CIS-Fatigue; Table S3: Spearman rank correlation coefficients of demographical, health status and disease-specific characteristics with disease-specific QoL in asthmatic patients (*n* = 653), Table S4: Multiple stepwise regression analysis of the significant variables associated with AQLQ.
