# Supplementary material for: Fatigue is Highly Prevalent in Patients with Asthma and Contributes to the Burden of Disease
Source: J Clin Med. 2018 Nov 23;7(12):471. doi: 10.3390/jcm7120471 (PMC6306949; doi:10.3390/jcm7120471)
Supplement: Supplementary file 1 [file jcm-07-00471-s001.pdf]

Online Data Supplement

Title

Fatigue is highly prevalent in patients with asthma and contributes to the burden of disease

Authors

Maarten Van Herck, Martijn A. Spruit, Chris Burtin, Remco Djamin, Jeanine Antons, Yvonne M.J. Goertz, Zjala Ebadi, Daisy J.A. Janssen, Jan H. Vercoulen, Jeannette B. Peters, Melissa S.Y. Thong, Jacqueline Otker, Arnold Coors, Mirjam A.G. Sprangers, Jean W.M. Muris, Emiel F.M. Wouters, and Alex van 't Hul

Table S1. Spearman rank correlation coefficients of demographical, health status and disease-specific characteristics with fatigue in asthmatic patients (n = 733).

	CIS-Fatigue (p)	
**Demographical, health status and disease-specific characteristics**	**Correlation coefficient**	**p-value**
Gender (male)	ρ 0.139	**< 0.001**
Age (years)	ρ -0.115	**0.002**
Weight (kg)	ρ 0.030	0.414
Waist circumference [a] (cm)	ρ 0.087	0.022
BMI (kg/m^2)	ρ 0.132	**< 0.001**
Exacerbations last 12 months [b] (n)	ρ 0.200	**< 0.001**
Hospitalization last 12 months [c] (n)	ρ 0.091	0.026
Smoking status [d] (current smoker)	ρ -0.030	0.432
Level of education [e] (secondary general education or higher)	ρ 0.145	**< 0.001**
Employment status [f] (paid work)	ρ -0.007	0.848
6MWT[g] (m)	ρ -0.243	**< 0.001**
FEV1 (L)	ρ -0.083	0.025
FVC (L)	ρ -0.158	**< 0.001**
FEV1/VC*100 (%)	ρ 0.133	**< 0.001**
CCI [h] (p)	ρ 0.070	0.162
MRC-Dyspnea [i] (p)	ρ 0.435	**< 0.001**
AQLQ [j] (p)	ρ -0.554	**< 0.001**
AQLQ symptoms [j] (p)	ρ -0.477	**< 0.001**
AQLQ activity limitation [j] (p)	ρ -0.591	**< 0.001**
AQLQ emotional function [j] (p)	ρ -0.361	**< 0.001**
AQLQ enviromental exposure [j] (p)	ρ -0.345	**< 0.001**
ACQ [k] (p)	ρ 0.455	**< 0.001**

P-value in bold indicates a significant association with fatigue (p<0.01). Alphabetic characters in superscript indicates a sample size deviant from n = 733 with: a. n = 696, b. n = 631, c. n = 601, d. n = 684, e. n = 686, f. n = 676, g. n = 652, h. n = 397, i. n = 532, j. n = 653, and k. n = 664. Abbreviations: AQLQ: Asthma Quality of Life Questionnaire; ACQ: Asthma Control Questionnaire; BMI: Body Mass Index; CIS-Fatigue: Checklist Individual Strength-Fatigue; CCI: Charlson Comorbidity Index; FEV1: Forced Expiratory Volume in one second; FEV1/VC*100: Tiffeneau index; FVC: Forced Vital Capacity; n: number of

subjects; p: points; MRC-Dyspnea: Medical Research Council-Dyspnea; QoL: Quality of life; 6MWT: Six-Minute Walk Test.

Table S2. Multiple stepwise regression analysis of the significant variables associated with CIS-Fatigue.

Model	Adjusted R^2	Predictors	Unstandardized coefficients		Standardized coefficients βeta	t-value	Significant value
			B	SE			
1.	0.210	(Constant)	27.783	1.259		22.071	< 0.001
		ACQ	6.205	0.613	0.461	10.116	< 0.001
2.	0.275	(Constant)	25.118	1.287		19.510	< 0.001
		ACQ	4.280	0.672	0.318	6.373	< 0.001
		MRC-Dyspnea	2.978	0.503	0.295	5.915	< 0.001
3.	0.289	(Constant)	29.485	1.982		14.873	< 0.001
		ACQ	4.103	0.668	0.305	6.141	< 0.001
		MRC-Dyspnea	3.288	0.510	0.326	6.445	< 0.001
		Age	-0.098	0.034	-0.127	-2.877	0.004

Abbreviations: ACQ: Asthma Control Questionnaire; CIS-Fatigue: Checklist Individual Strength-Fatigue; MRC-Dyspnea: Medical Research Council-Dyspnea; SE: Standard Error.

Table S3. Spearman rank correlation coefficients of demographical, health status and disease-specific characteristics with disease-specific QoL in asthmatic patients (n = 653).

	AQLQ (p)	
**Demographical, health status and disease-specific characteristics**	**Correlation coefficient**	**p-value**
Gender (male)	ρ -0.090	0.021
Age (years)	ρ 0.115	**< 0.001**
Weight (kg)	ρ -0.071	0.070
Waist circumference [a] (cm)	ρ -0.102	0.011
BMI (kg/m^2)	ρ -0.194	**< 0.001**
Exacerbations last 12 months [b] (n)	ρ -0.279	**< 0.001**
Hospitalization last 12 months [c] (n)	ρ -0.049	0.251
Smoking status [d] (current smoker)	ρ -0.031	0.450
Level of education [e] (secondary general education or higher)	ρ -0.156	**< 0.001**
Employment status [f] (paid work)	ρ -0.011	0.793
CIS-Fatigue (p)	ρ -0.554	**< 0.001**
6MWT [g] (m)	ρ 0.287	**< 0.001**
FEV1 (L)	ρ 0.072	0.067
FVC(L)	ρ 0.161	**< 0.001**
FEV1/VC*100 (%)	ρ -0.162	**< 0.001**
CCI [h] (p)	ρ -0.079	0.132
MRC-Dyspnea [i] (p)	ρ -0.488	**< 0.001**
ACQ [j] (p)	ρ -0.777	**< 0.001**

P-value in bold indicates a significant association with AQLQ ($p < 0.01$). Alphabetic characters in superscript indicates a sample size deviant from $n = 653$ with: a. $n = 620$, b. $n = 574$, c. $n = 547$, d. $n = 612$, e. $n = 621$, f. $n = 610$, g. $n = 585$, h. $n = 361$, i. $n = 484$, and j. $n = 641$. Abbreviations: AQLQ: Asthma Quality of Life Questionnaire; ACQ: Asthma Control Questionnaire; BMI: Body Mass Index; CIS-Fatigue: Checklist Individual Strengths-Fatigue; CCI: Charlson Comorbidity Index; FEV1: Forced Expiratory

Volume in one second; FEV1/VC*100: Tiffeneau-index; FVC: Forced Vital Capacity; n: number of subjects; p: points; MRC-Dyspnea: Medical Research Council-Dyspnea; QoL: Quality of life; 6MWT: Six-Minute Walk Test.

Table S4. Multiple stepwise regression analysis of the significant variables associated with AQLQ.

Model	Adjusted R^2	Predictors	Unstandardized coefficients		Standardized coefficients	t-value	Significant value
			B	SE	βeta		
1.	0.298	(Constant)	6.920	0.171		40.370	< 0.001
		CIS-Fatigue	-0.053	0.004	-0.547	-12.750	< 0.001
2.	0.384	(Constant)	7.014	0.161		43.532	< 0.001
		CIS-Fatigue	-0.039	0.004	-0.397	-8.778	< 0.001
		MRC-Dyspnea	-0.322	0.044	-0.331	-7.338	< 0.001
3.	0.410	(Constant)	7.009	0.158		44.461	< 0.001
		CIS-Fatigue	-0.035	0.004	-0.362	-8.053	< 0.001
		MRC-Dyspnea	-0.319	0.043	-0.328	-7.427	< 0.001
		Exacerbations in the last 12 months	-0.133	0.031	-0.170	-4.230	< 0.001
4.	0.422	(Constant)	6.552	0.218		30.085	< 0.001
		CIS-Fatigue	-0.033	0.004	-0.339	-7.518	< 0.001
		MRC-Dyspnea	-0.348	0.044	-0.359	-7.989	< 0.001
		Exacerbations in the last 12 months	-0.135	0.031	-0.173	-4.349	< 0.001
		Age	0.009	0.003	0.121	3.011	0.003

Abbreviations: AQLQ: Asthma Quality of Life Questionnaire; CIS-Fatigue: Checklist Individual Strength-Fatigue; MRC-Dyspnea: Medical Research Council-Dyspnea; SE: Standard Error.
